# Low to moderate prenatal alcohol exposure and neurodevelopment in a prospective cohort of early school aged children

**DOI:** 10.1038/s41598-024-57938-7

**Published:** 2024-03-27

**Authors:** Evelyne Muggli, Jane Halliday, Stephen Hearps, Thi-Nhu-Ngoc Nguyen, Anthony Penington, Deanne K. Thompson, Alicia Spittle, Della A. Forster, Sharon Lewis, Elizabeth J. Elliott, Peter J. Anderson

**Affiliations:** 1 Murdoch Children’s Research Institute, Parkville, VIC Australia; 2 Department of Paediatrics, The University of Melbourne, Melbourne, VIC Australia; 3 Royal Children’s Hospital, Parkville, VIC Australia; 4 Turner Institute for Brain and Mental Health, School of Psychological Sciences, Monash University, Clayton, VIC 3800 Australia; 5 Department of Physiotherapy, The University of Melbourne, Melbourne, VIC Australia; 6 Judith Lumley Centre, School of Nursing and Midwifery, SHE College, La Trobe University, Melbourne, VIC Australia; 7 The Royal Women’s Hospital, Parkville, VIC Australia; 8 Child and Adolescent Health, Faculty of Medicine and Health, The University of Sydney, Sydney, NSW Australia; 9 Kid’s Research, Sydney Children’s Hospitals Network, Westmead, Sydney, NSW Australia

**Keywords:** Prenatal alcohol exposure, Neurodevelopment, Observational epidemiology, Cohort studies, Child development, Epidemiology, Paediatric research

## Abstract

Evidence is strong for adverse fetal effects of high level or chronic prenatal alcohol exposure (PAE), but many pregnant women continue to drink at lower levels. The ‘Asking Questions about Alcohol in pregnancy’ prospective cohort aimed to determine the neurodevelopmental consequences at 6–8 years of age of low to moderate PAE. 1570 women from seven public antenatal clinics in Melbourne, Australia, provided information on frequency and quantity of alcohol use, and obstetric, lifestyle and socio-environmental confounders at four gestation timepoints. PAE was classified into five trajectories plus controls. At 6–8 years, 802 of 1342 eligible families took part and completed a questionnaire (60%) and 696 children completed neuropsychological assessments (52%). Multiple linear regressions examined mean outcome differences between groups using complete case and multiple imputation models. No meaningful relationships were found between any of the PAE trajectories and general cognition, academic skills, motor functioning, behaviour, social skills, social communication, and executive function. Maternal education most strongly influenced general cognition and academic skills. Parenting behaviours and financial situation were associated with academic skills, behaviour, social skills and/or executive function. The lack of association between PAE and neurodevelopment at 6–8 years may partly be explained by cumulative positive effects of socio-environmental factors.

## Introduction

Fetal Alcohol Spectrum Disorder (FASD) is an umbrella term for a broad spectrum of neurodevelopmental and physical deficits observed in children, adolescents, and adults with a history of episodic or chronic alcohol exposure before birth^1,2^. The severity of FASD symptoms is related to the level, pattern, and timing of prenatal alcohol exposure (PAE)^3^. However, despite numerous epidemiological investigations and systematic reviews spanning several decades, the nature of the dose–response relationship between PAE and fetal effects remains unclear^4^. Specifically, there is a high degree of individual variation in the susceptibility to harm for fetuses exposed to similar prenatal alcohol patterns, likely due to genetic, metabolic, nutritional, social, and environmental factors^5,6^. Thus, research to date has failed to establish a safe level of alcohol a woman can consume during different stages of pregnancy^4,7^ and health policies around the world largely recommend abstinence as the safest option. However, many women continue to drink some alcohol while pregnant, especially around the time of conception and before they know of the pregnancy^8–10^. The lack of convincing evidence of harm from lower levels of PAE,^11,12^ and conflicting messages from health professionals concerning adverse effects of low to moderate PAE on the fetus are reasons given by some women for their decision not to abstain^13,14^. Human research to date has provided mixed evidence on the potential effects of low to moderate PAE on child neurodevelopmental outcomes, with a recent systematic review reporting adverse effects in six studies, no effect in five studies, and a weak positive effect in two^15^. The conflicting findings of these studies may in part be due to limitations in exposure measurement, a lack of sensitivity for detecting impairments for some neurodevelopmental outcomes, and inadequate accounting for confounding by environmental and social factors^4,15^.

Over time, methods used to quantify PAE were variable and mostly categorised as risk groupings with pre-defined cut off scores. Such groupings lacked the detail necessary to draw conclusions about a potential dose–response relationship and made comparison across studies very difficult^4^.

The Asking Questions about Alcohol in Pregnancy (AQUA) prospective cohort study was designed to assess short- and long-term effects of common drinking patterns in pregnancy while incorporating a systematic measurement of self-reported PAE and collecting information on a wide range of confounders across development^16^. In order to represent real-life, unit-level PAE, group-based trajectory modelling was used to identify six consumption trajectories which also incorporated four timepoints across gestation^17^. Of 1570 women taking part in the AQUA study, 59% reported drinking alcohol during pregnancy and 19% reported at least one episode of binge drinking prior to pregnancy recognition^8^. Using 3-dimensional craniofacial imaging, an association was found between low to moderate PAE and facial shape in the offspring at 12 months of age, with differences from non-exposed controls concentrated around the nose, eyes, and mouth^18^. While the craniofacial phenotype observed was reminiscent of that seen in FASD, any potential clinical significance of these findings is yet to be determined. At 2 years of age, no adverse association was detected between low to moderate PAE and child neurodevelopment using the Bayley Scales of Infant and Toddler Development (Bayley-III)^19^.

However, early developmental assessments are only moderately predictive of later outcomes^20^ and neurodevelopment is an ongoing process. Impairments in specific domains (e.g. language, executive function) can only be identified following the emergence of these abilities. The present study reassessed children in the AQUA cohort at 6–8 years of age with the aim to determine the long-term neurodevelopmental consequences of low to moderate levels or sporadic prenatal alcohol consumption^21^.

We hypothesised that PAE is associated with subtle neuropsychological deficits in motor, attention, executive function, memory and/or behaviour domains at 6–8 years of age and that PAE associations are influenced by the timing and quantity of alcohol exposure, individual child and maternal characteristics, and socio-environmental factors.

## Methods

Participants were children aged 6–8 years born to mothers originally recruited into the AQUA cohort in in 2011/2012^16^. Of the 1570 mother and child dyads from the original cohort, 55 mothers had withdrawn from the study. We excluded 108 who were lifetime alcohol abstainers because our target population was children of mothers who normally drink some alcohol. Another 59 mothers were excluded who could not be classified because they abstained in the first trimester, then averaged an intake of less than one standard drink per week for the remainder of their pregnancy. Therefore, for the current study, 1348 mothers and children were invited to participate. Following the invitation to take part, a further six families were excluded, because of a recent oncology diagnosis in the child (n = 3) or because of a later diagnosed condition impacting long-term development (one child with Down syndrome, one child with Dopa Responsive Dystonia and another child with Sanfilippo Syndrome)^21^. The final number of families eligible to participate was 1342, of whom 802 consented (60%) and participated in aspects of the follow-up from June 2018 to April 2021. Neuropsychological assessment data were available for 696 children (52%). Five hundred and forty eligible families did not take part: 308 who opted out; 71 for whom we had no current contact details; and 161 who opted out passively either by not responding to any of our follow-ups or after initially expressing interest. Mothers of participating children were less likely to have been abstinent in pregnancy, smokers in pregnancy, or under 30 years of age at the time of birth than non-participants. They were more likely to be tertiary educated at the time of initial recruitment and of Caucasian/white ethnicity^21^.

### Prenatal alcohol exposure (PAE) assessment

Information on alcohol consumption was collected in the original AQUA study via three questionnaires administered in pregnancy^8^. The first questionnaire was completed on paper at the time of recruitment. There was a choice between paper and electronic versions for subsequent questionnaires with around two thirds taking up the latter. All questionnaires were self-completed.

#### Timing of exposure

Maternal alcohol consumption data covered four stages of pregnancy: (1) trimester one pre-pregnancy aware; (2) trimester one post-pregnancy aware; (3) trimester two; and (4) trimester three.

#### Levels of exposure

Women were asked to use a pictorial drinks guide, listing common types and volumes of alcoholic drinks, to identify their ‘usual’ pattern of drinking, with provision for up to five types of alcoholic drink. For each beverage identified, they were asked how often they usually drank this type of alcohol and how many drinks they usually consumed on each occasion. Women were also asked if there were any ‘special occasions’ (or difficult times) when they consumed more alcohol than usual, the frequency of these occasions, the drink types, and the number of drinks per occasion. Estimates from ‘special occasions’ were combined with information from ‘usual’ alcohol consumption^8^. The number and types of drink reported by women were converted to grams of absolute alcohol consumed for each drink, summed and averaged over one week (gAA/week).

To account for timing, dose and duration of alcohol exposure, we used Group-based Trajectory Modelling (GBTM) to classify unit-level consumption data (gAA/week) at four timepoints across gestation into six alcohol consumption trajectories. Labels were assigned based on consumption in trimester one, before and after pregnancy awareness, and whether alcohol use was continued throughout pregnancy^17^. Participant exposures are illustrated in Table 1.

**Table 1:** Numbers of participants and maternal prenatal weekly alcohol consumption by exposure trajectory.

Exposure trajectory	Description	N (%)
Abstained/control	No alcohol consumption during pregnancy	257 (32.0)
Low discontinued	Median alcohol consumption of 3gAA/week and none post pregnancy recognition i.e. approximately one to two standard drinks^a^, once or twice per month prior to pregnancy recognition	127 (15.8)
Moderate discontinued	Median alcohol consumption of 35gAA/week (i.e. approximately three standard drinks per week ) and none post first trimester	91 (11.4)
Low sustained	Continued low median consumption post-awareness	92 (11.5)
Moderate sustained	Median consumption of 35gAA/week prior to pregnancy recognition and a continued low consumption trend post-awareness	209 (26.1)
High sustained	Median consumption of 190gAA/week prior to pregnancy recognition (i.e. approximately three to four standard drinks, three to four times a week) and a continued moderate consumption trend post-awareness	26 (3.2)

^a^One Australian standard drink is equivalent to 10 g of absolute alcohol.

### Neuropsychological assessment

When possible, neuropsychological clinical assessments were performed at the Murdoch Children’s Research Institute in Melbourne, Australia. This paper focuses on general cognition, executive function, academic skills, motor skills and behavior/social skills, all of which are relevant to FASD diagnostic guidelines^22,23^. Selected measures are widely used in clinical and research settings and are well validated^22,24^. Assessments were conducted by trained assessors who were blinded to PAE and previous child assessments. Due to the COVID-19 pandemic, the recruitment period included two government-mandated, state-wide lockdowns in Victoria, Australia. As a result, adaptations to the assessment procedures were necessary to comply with institutional and government guidelines for a safe environment for study participants and assessors. From June 2020, 169 of the assessments were conducted via telehealth and another 73 in-person with physical distancing practices in place. Test results were also obtained for nine children who had a recent clinical assessment performed externally^21^. Primary caregivers (i.e. AQUA study mother in most cases) also completed a questionnaire online to rate their child’s emotional and behavioural status and movement and coordination.

1. *General cognition* Core subtests of the Wechsler Intelligence Scale for Children (WISC-V Australian & New Zealand Standardised Edition) were administered to estimate full-scale IQ^25^. Certain subtests were not able to be administered via telehealth (i.e., Block Design and processing speed subtests) and/or with physical distancing in place (i.e., Block Design). In these cases Full-scale IQ was imputed with the Non-Motor Full Scale Score (NMFSS) (n = 170) in accordance with publishers guidelines for coding WISC-V telehealth assessments^26^, but the visual spatial and processing speed indices were missing.
2. *Academic skills* Subtests from the Wechsler Individual Achievement Test (WIAT-III Australian & New Zealand Standardised Edition) were used to assess components of academic skills. Literacy was assessed using the ‘Word Reading’ and ‘Spelling’ subtests, while mathematics was assessed using ‘Numerical Operations’^27^. ‘Numerical Operations’ was not able to be assessed via telehealth.
3. *Motor Functioning* The Movement Assessment Battery for Children (MABC2) was administered to assess motor functioning (Manual Dexterity, Aiming and Catching, Balance)^28^. The MABC2 was not able to be administered via telehealth. Parents also completed the Developmental Coordination Disorder Questionnaire (DCDQ07, total score)^29^.
4. *Behaviour, social skills and social communication* Parents completed the Strengths & Difficulties Questionnaire (SDQ)^30^, with scales assessing emotional symptoms, conduct problems, hyperactivity/inattention, peer problems, and prosocial behaviour. The Social Communication Questionnaire (SCQ)^31^ was also completed by parents to assess symptoms associated with autism spectrum disorder.
5. *Executive function* Parents rated their children’s executive function in everyday settings on the Behavior Rating Inventory of Executive Function-Second Edition (BRIEF-2)^32^. T-scores of the Global Executive Composite [GEC], Cognitive Regulation Index [CRI]), Behavior Regulation Index [BRI]), and the Emotion Regulation Index [ERI]) were examined.

### Potential confounders

Testing modality (telehealth or in-person assessment), child sex and age at assessment, as well as extensive demographic and socio-environmental factors that may confound or modify the relationship between PAE and child outcomes were included (Table 2, Supplementary Table 1).

**Table 2:** Cohort characteristics by prenatal alcohol trajectory.

		Abstained/control		Low discontinued		Moderate discontinued		Low sustained		Moderate sustained		High sustained	
		n = 257		n = 127		n = 91		n = 92		n = 209		n = 26	
		n	(%)	n	(%)	n	(%)	n	(%)	n	(%)	n	(%)
Assessment type	*Full assessment in clinic*	152	(59.1)	76	(59.8)	43	(47.3)	54	(58.7)	112	(53.6)	8	(30.8)
	*Covid modified in clinic*	21	(8.2)	12	(9.5)	6	(6.6)	6	(6.5)	25	(12.0)	3	(11.5)
	*Covid modified online (telehealth)*	47	(18.3)	23	(18.1)	21	(23.1)	21	(22.8)	46	(22.0)	11	(42.3)
	*External test scores*	3	(1.2)	0	(0.0)	1	(1.1)	1	(1.1)	3	(1.5)	1	(3.9)
	*Questionnaire only*	34	(13.2)	16	(12.6)	20	(22.0)	10	(10.9)	23	(11.0)	3	(11.5)
Child sex	*Female*	121	(47.1)	75	(59.1)	46	(50.6)	41	(44.6)	96	(45.9)	13	(50.0)
Child ethnicity^a^	*White/Caucasian*	185	(72.3)	98	(79.0)	71	(79.8)	79	(87.8)	187	(90.8)	26	(100.0)
Parent indicated child diagnosis	*Yes*	13	(5.1)	4	(3.2)	6	(6.6)	2	(2.2)	12	(5.7)	0	(0.0)
Birth order	S*econd-born or higher*	136	(53.8)	54	(43.2)	40	(44.4)	60	(66.7)	104	(49.8)	12	(46.2)
Small for gestational age	*Yes*	19	(7.6)	10	(7.9)	7	(7.9)	10	(11.1)	3	(1.5)	1	(3.9)
Preterm birth	*Yes*	15	(6.0)	9	(7.1)	4	(4.5)	0	(0.0)	6	(2.9)	1	(3.9)
Planned pregnancy	*Yes*	209	(81.6)	97	(77.0)	59	(64.8)	68	(74.7)	166	(79.4)	23	(88.5)
Maternal pre-pregnancy binge episode^a^	*Yes*	40	(15.6)	24	(18.9)	79	(86.8)	0	(0.0)	172	(82.3)	24	(92.3)
Pre-pregnancy BMI^a^	*Normal weight*	141	(56.2)	83	(66.9)	43	(50.0)	53	(59.6)	141	(70.2)	17	(65.4)
	*Underweight*	11	(4.4)	5	(4.0)	2	(2.3)	8	(9.0)	7	(3.5)	0	(0.0)
	*Overweight*	42	(16.7)	17	(13.7)	27	(31.4)	20	(22.5)	32	(15.9)	6	(23.1)
	*Obese*	57	(22.7)	19	(15.3)	14	(16.3)	8	(9.0)	21	(10.5)	3	(11.5)
Maternal pre-pregnancy alcohol feel rate^a^	*Quickly*	135	(53.4)	65	(52.0)	27	(29.7)	45	(48.9)	63	(30.1)	8	(30.8)
Maternal smoking during pregnancy^a^	Yes	31	(12.1)	11	(8.7)	18	(19.8)	7	(7.6)	46	(22.0)	5	(19.2)
Maternal current smoking	Yes	19	(7.5)	6	(4.8)	13	(14.8)	6	(6.7)	20	(9.7)	4	(16.0)
Family structure	*Nuclear, dual caregiver*	218	(86.5)	112	(88.9)	79	(89.8)	74	(83.2)	187	(90.3)	24	(96.0)
	*Separated, shared custody*	18	(7.1)	8	(6.4)	2	(2.3)	10	(11.2)	17	(8.2)	1	(4.0)
	*Sole parent*	16	(6.4)	6	(4.8)	7	(8.0)	5	(5.6)	3	(1.5)	0	(0.0)
Maternal education^a^	*High school*	34	(13.7)	6	(4.8)	13	(14.9)	3	(3.4)	20	(9.7)	2	(8.0)
	*Trade/diploma*	71	(28.5)	27	(21.4)	30	(34.5)	21	(23.6)	33	(16.0)	3	(12.0)
	*Tertiary*	144	(57.8)	93	(73.8)	44	(50.6)	65	(73.0)	153	(74.3)	20	(80.0)
Financial situation	*Doing alright*	123	(48.8)	59	(46.8)	42	(47.7)	41	(46.6)	88	(42.5)	9	(36.0)
	*Living comfortably*	86	(34.1)	52	(41.3)	35	(39.8)	38	(43.2)	94	(45.4)	11	(44.0)
	*Finding it difficult*	43	(17.1)	15	(11.9)	11	(12.5)	9	(10.2)	25	(12.1)	5	(20.0)

		M	(SD)	M	(SD)	M	(SD)	M	(SD)	M	(SD)	M	(SD)
Child age at assessment	*Years*	7.3	(0.6)	7.3	(0.6)	7.5	(0.6)	7.4	(0.6)	7.4	(0.6)	7.6	(0.4)
Socio-economic background	*Z-scored*	0.31	(0.69)	0.44	(0.60)	0.4	(0.61)	0.38	(0.75)	0.36	(0.76)	0.48	(0.77)
Maternal age at birth	*Years*	32.4	(4.7)	32.4	(4.6)	32.7	(4.9)	33.5	(3.8)	32.7	(4.0)	34.6	(3.6)
Maternal current alcohol use^a^	S*ummary score*	1.6	(1.5)	2.4	(1.5)	3.2	(2.4)	2.7	(1.6)	4.0	(1.9)	5.9	(2.7)
Maternal Depression	S*ummary score*	4.5	(6.3)	4.6	(6.3)	4.5	(5.7)	4.4	(5.4)	4.3	(5.6)	5.3	(5.5)
Maternal Anxiety	S*ummary score*	4.1	(5.5)	2.4	(3.7)	3.1	(4.8)	3.3	(4.8)	3.2	(4.2)	3.3	(3.7)
Maternal Stress	S*ummary score*	10.2	(7.6)	9.4	(7.3)	9.2	(7.5)	10.2	(8.3)	10.5	(7.1)	11.7	(6.2)
Parenting style	*Warmth, summary score*	4.4	(0.5)	4.3	(0.5)	4.5	(0.5)	4.4	(0.5)	4.3	(0.5)	4.4	(0.5)
	*Control, summary score*	3.8	(0.7)	3.9	(0.6)	3.8	(0.6)	3.8	(0.6)	3.8	(0.6)	3.7	(0.5)
	*Irritability, summary score*	2.5	(0.6)	2.4	(0.6)	2.5	(0.6)	2.6	(0.5)	2.6	(0.6)	2.8	(0.6)
General family functioning	S*ummary score*	1.5	(0.4)	1.5	(0.5)	1.5	(0.4)	1.6	(0.4)	1.6	(0.4)	1.7	(0.5)
Child special health care needs	*Dependency on prescription medication*	25.0	(9.8)	17.0	(13.4)	14.0	(15.4)	9.0	(9.8)	24.0	(11.5)	1.0	(3.9)

^a^Pearson Chi Square for between group differences is *p* < 0.01.

### Statistical analysis

Descriptive statistics were generated to summarise characteristics of alcohol trajectory groups, using means and standard deviations for continuous measures, and frequencies and proportions for categorical variables. Analysis of variance (ANOVA) models compared between-group means, and chi-squared tests compared proportions.

Data missingness was small, being 0.85% overall, and the maximum amount within any variable being 3.74%. To handle this missingness, multiple imputation was performed on the eligible sample using chained equations and 50 replications, where the imputation was carried out by recruitment modality (full assessment/telehealth/questionnaire).

Linear regressions examined mean outcome differences between PAE groups with unexposed controls being the reference group. Putative covariates were first explored independently in relation to each outcome (results presented as supplemental data). Given the number of covariates considered, a *p* value of < 0.01 was chosen as cut-off for inclusion in multiple regression models alongside PAE groups. Model-estimated group means were generated and plotted with 95% confidence intervals (95% CI) to examine the relationship between control and PAE groups for each outcome (full model statistics are presented as supplemental data). All analyses were carried out using Stata v17.0 and employed a significance level of *p* < 0.05 (unless specified otherwise).

### Ethics approval

The AQUA study has approval from the Human Research Ethics Committee of the Royal Children’s Hospital, Melbourne, Australia (approval numbers #38025 & # 31055). Informed consent for participation was obtained from a parent (mostly the mother) and all methods were carried out in accordance with relevant guidelines and regulations, specifically, the National Statement on Ethical Conduct in Human Research (2007) issued by the National Health and Medical Research Council of Australia.

## Results

Child, maternal and family characteristics across exposure trajectories were similar between the groups, but there was a greater tendency for children in the moderate and high exposure trajectories to be of Caucasian/white ethnicity. Higher percentages of mothers in the moderate and high exposure trajectories had a tertiary education, smoked in pregnancy, and a lower frequency of a pre-pregnancy body mass index indicating obesity. Mothers of children in the moderate and high exposure trajectories also tended to self-report a lower rate of feeling the effects of alcohol quickly, at least one pre-pregnancy binge drinking episode, and higher current alcohol use (Table 2).

### Bivariate analyses

Results from the complete case and multiple imputed bivariate analyses are presented as Supplementary Tables 2 and 3. Inclusion of cofactors in the multivariate analyses varied for each outcome. Some of the key factors identified as potential confounders and included in the final analyses were child sex, child age at assessment, maternal pre-pregnancy body mass index (BMI), maternal educational attainment, and family financial situation. Maternal mental health, parenting style and general family functioning featured particularly in outcomes related to child behaviour and social skills.

### Multivariate analyses

**Figure 1:**
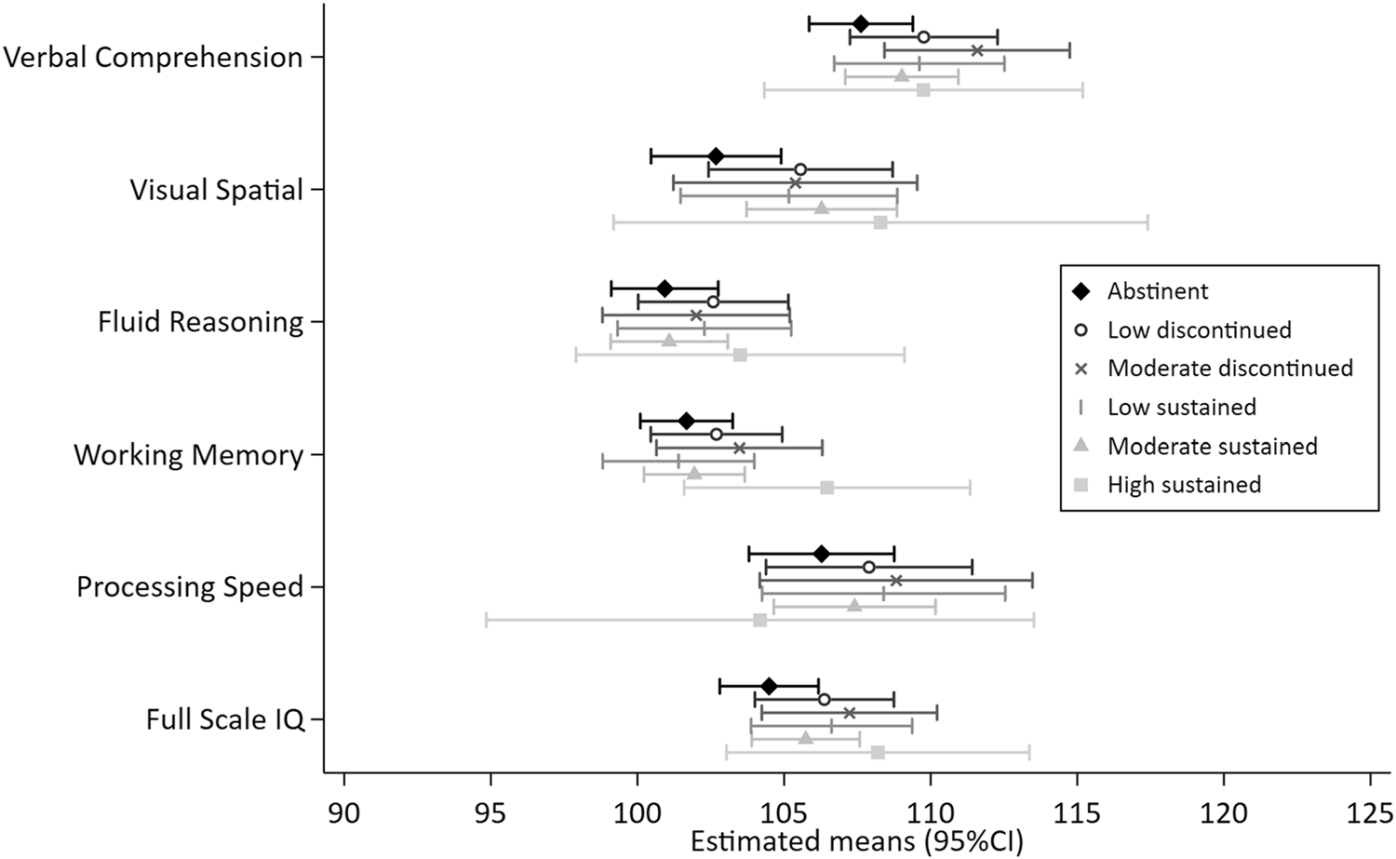
General cognition (Wechsler Intelligence Scale for Children, 5th Edition (WISC-V). Mean (SD) standard score of 100 (15).) at ages 6–8 years by prenatal alcohol exposure trajectory.

**Figure 2:**
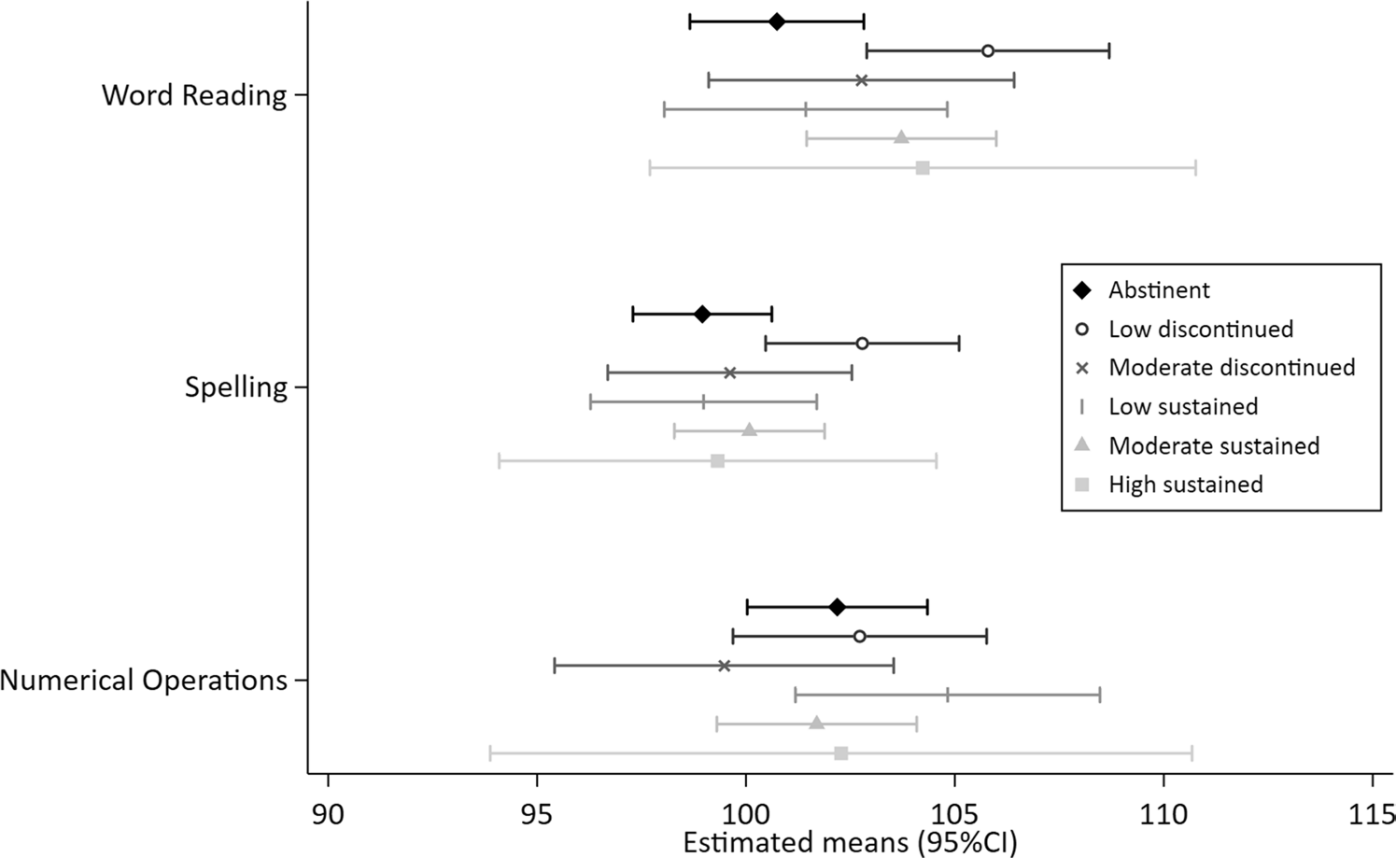
Academic skills (Wechsler Individual Achievement Test, 3rd Edition (WIAT-III). Mean (SD) standard score of 100 (15).) at ages 6–8 years by prenatal alcohol exposure trajectory.

**Figure 3:**
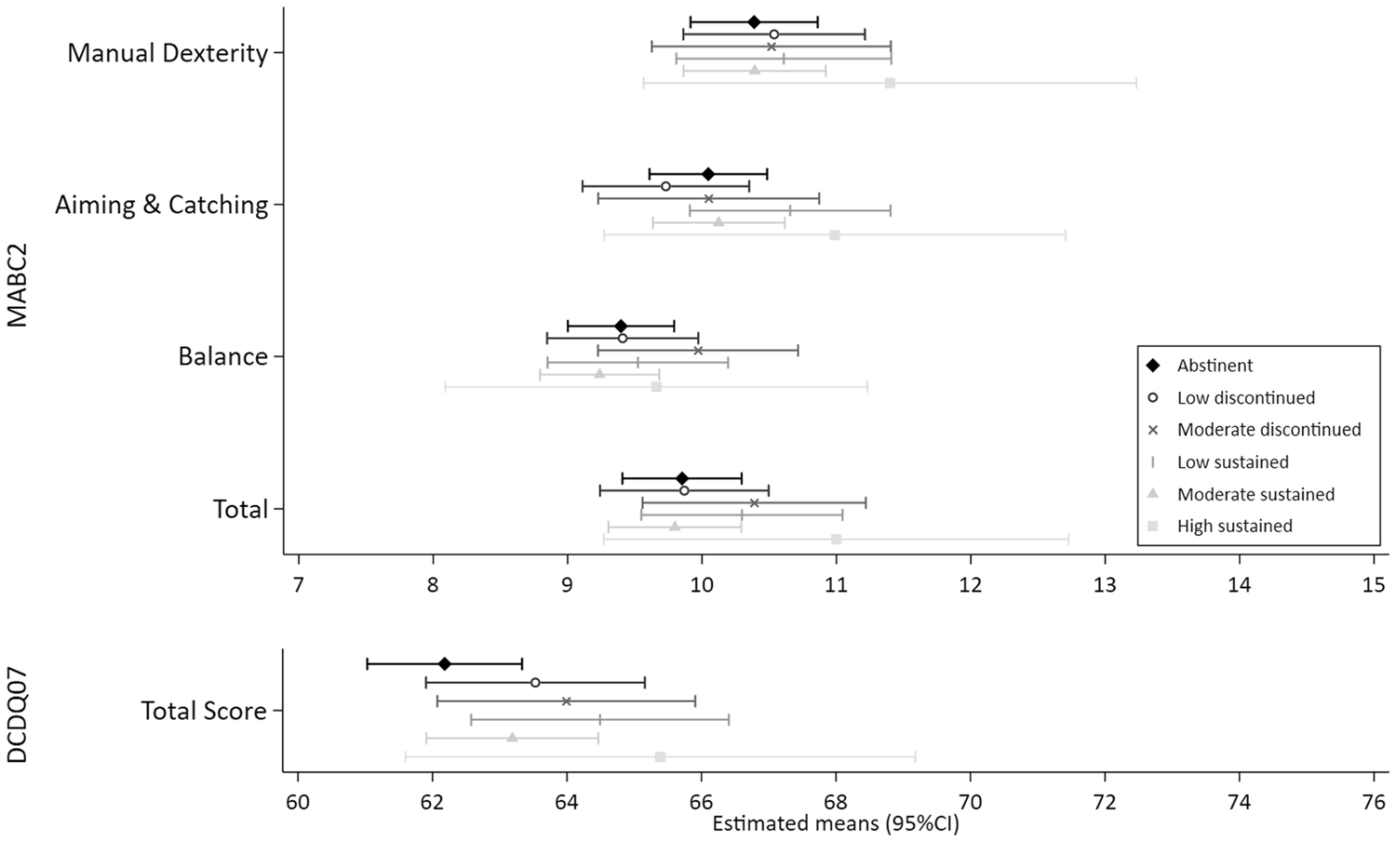
Motor function at ages 6–8 years by prenatal alcohol exposure trajectory. MABC2: Movement Assessment Battery for Children, 2nd Edition. Component and composite standard scores have a mean of 10. DCDQ07: Developmental Coordination Disorder Questionnaire 2007. Higher scores indicate better performance.

**Figure 4:**
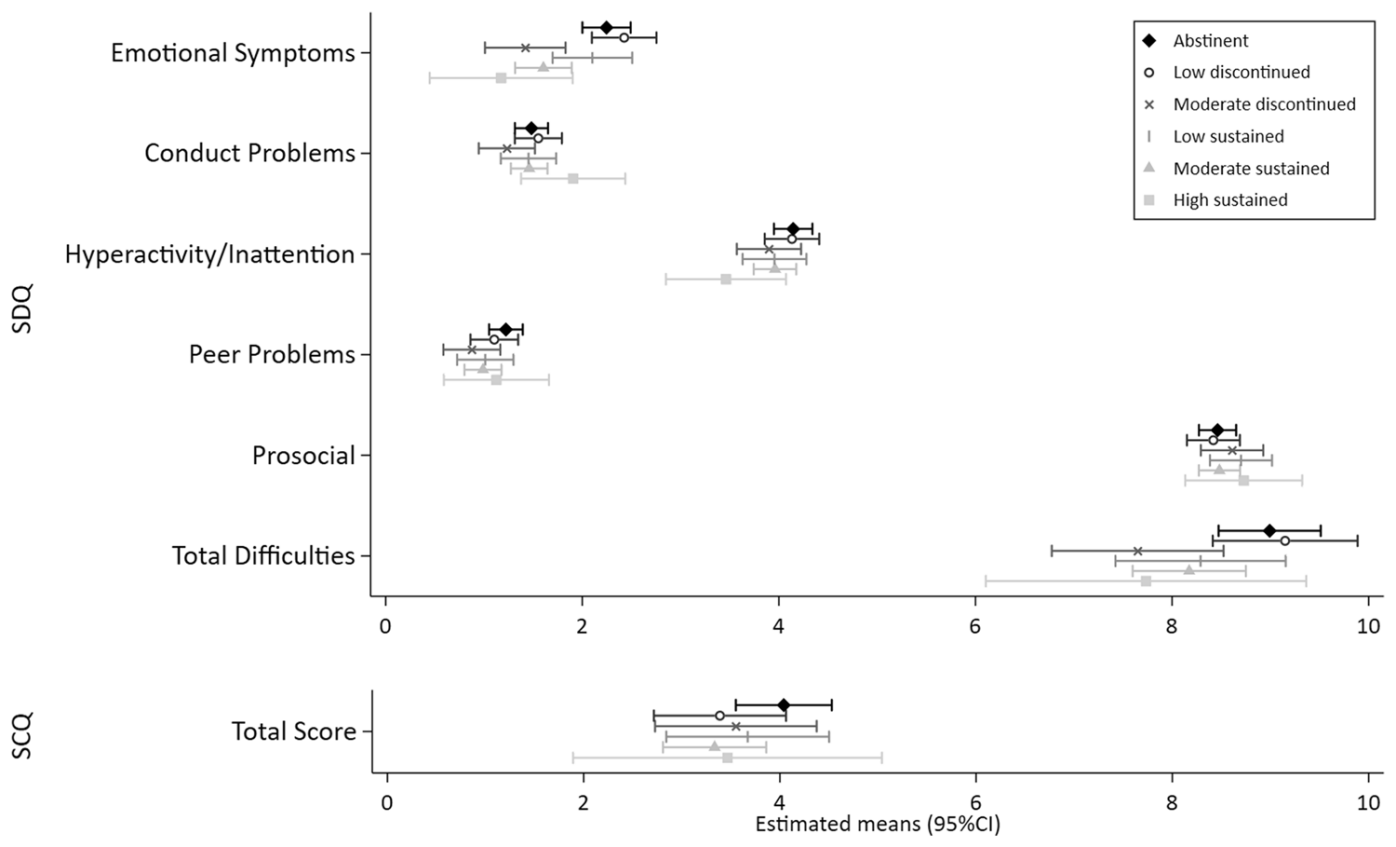
Behaviour, social skills & social communication at ages 6–8 years by prenatal alcohol exposure trajectory. SDQ: Strength and Difficulties Questionnaire. Lower scores indicate better performance, except for ‘Prosocial’ where a higher score indicates a better outcome. ‘Total Difficulties’ was generated from the sum of the four sub-scales of ‘Emotional Symptoms’, ‘Conduct Problems’, ‘Hyperactivity/Inattention’ and ‘Peer Problems’. SCQ: Social Communications Questionnaire. Higher scores represent more difficulties with social communication.

**Figure 5:**
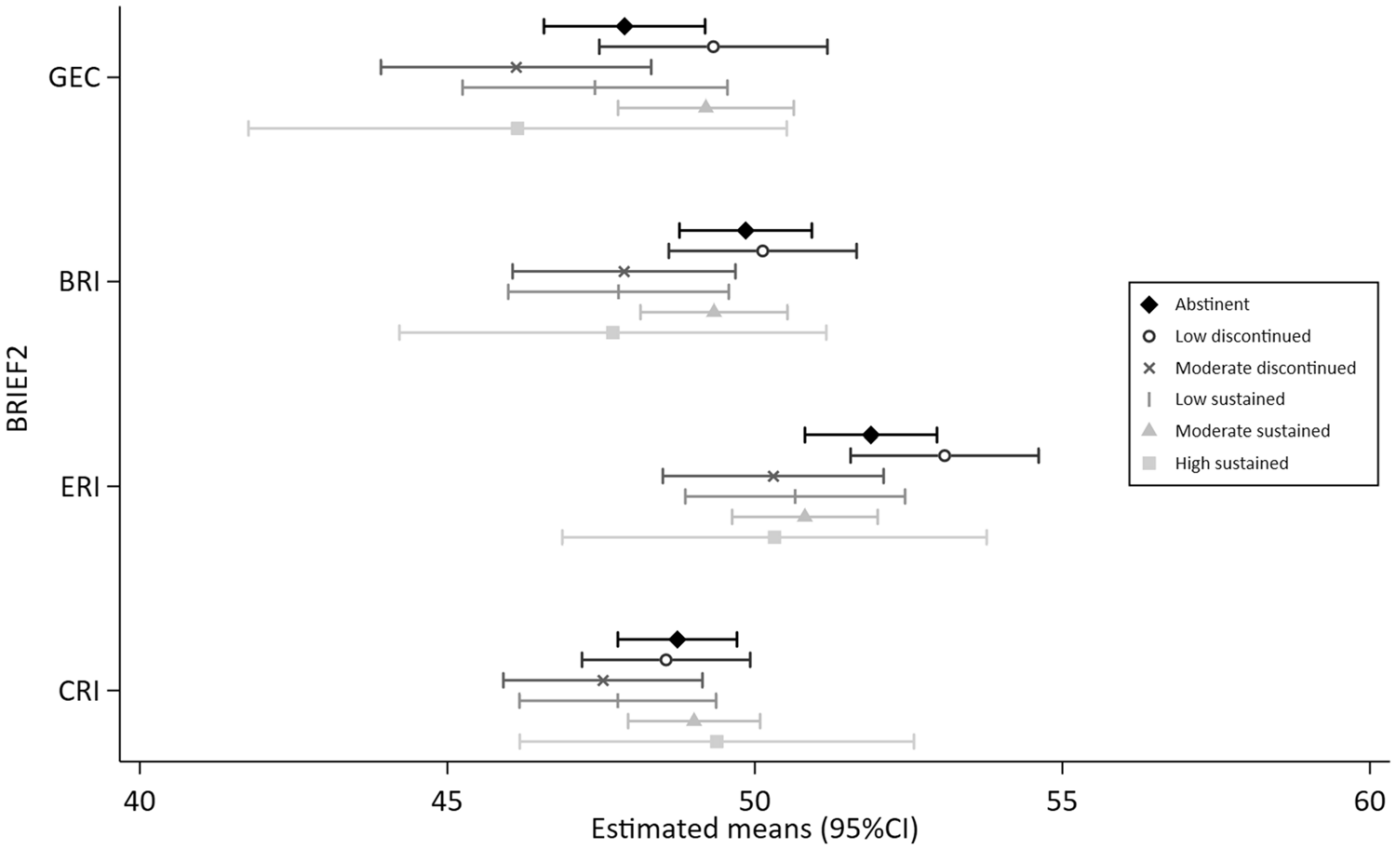
Executive function at ages 6–8 years by prenatal alcohol exposure trajectory. BRIEF-2: Behavior Rating Inventory of Executive Function, Second Edition; Global Executive Composite [GEC], Cognitive Regulation Index [CRI]), Behavior Regulation Index [BRI]), and Emotion Regulation Index [ERI]). Higher T scores are indicative of increasing executive function difficulties.

Figures 1, 2, 3, 4 and 5 show the MI estimated means and 95% confidence intervals in forest plots for each outcome grouped by domain: general cognition (Fig. 1), academic skills (Fig. 2), motor functioning (Fig. 3), behaviour, social skills & social communication (Fig. 4) and executive function (Fig. 5). Detailed results from the complete case models (CC) and the multiple imputation (MI) models are presented as supplementary material (Supplementary Tables 4–8 (MI) and 9–13 (CC). Results from the MI models are similar to the complete case models.

In general, 95% CIs of all PAE trajectories overlapped with the abstinent group across the 25 outcome measures. Compared to the unexposed control group, higher levels of performance (ie. lower levels of difficulties) were indicated for 10 outcomes with some exposure trajectories, but no discernible patterns were observed (Figs. 1, 2, 3, 4, 5 and Supplementary Tables 4–8): WISC verbal comprehension (moderate discontinued) and visual spatial (moderate sustained); WIAT word reading and spelling (low discontinued); DCDQ total score (low sustained); SDQ emotional symptoms (moderate discontinued, moderate sustained and high sustained), hyperactivity/inattention (high sustained), peer problems (moderate discontinued) and total difficulties (moderate discontinued, moderate sustained).

Independently of PAE, maternal education was strongly predictive of higher verbal comprehension, fluid reasoning, working memory, full scale IQ and all academic skills scores in the fully adjusted models. Financial difficulty was negatively associated with academic skills. Parenting behaviours^26^ were also correlated with some of the outcome measures. For example, parental control (e.g. setting and enforcing boundaries) was associated with higher academic skills and parental irritability (e.g. expressing disapproval, lack of praise) was consistently negatively associated with behaviour, social skills and executive function. A child’s dependency on prescription medications (Child Special Health Care Needs Screener)^33^ was negatively associated with 15 outcome measures (Supplementary Tables).

## Discussion

After applying a comprehensive assessment of PAE and including a wide range of confounders in our models, we found no evidence for a detrimental association between any of the PAE trajectories examined and child neurodevelopment in children aged 6–8 years. While there was an indication of better functioning with some of the PAE trajectories for a small number of outcomes investigated, no meaningful relationships between exposure and outcome were apparent and likely chance findings.

The latest review of the literature on low to moderate PAE and neurodevelopmental outcomes found that although a positive association with outcomes related to cognition was reported in several studies, most studies published null findings^7^. The authors of the review concluded, as many have before, that methodological differences and inadequacies continue to limit our ability to summarise and interpret the evidence with confidence.

A 2020 meta-analysis of six longitudinal cohorts in the United States using data pooled across 134 neurocognitive measures reported adverse effects of PAE on cognitive function, executive function (set-shifting), and reading and mathematical achievement. Median maternal alcohol consumption in pregnancy for 2236 participants averaged about 2.5 drinks per occasion on 3–4 days per week^34^. Although these findings contrast with ours, the exposure group most closely matching this alcohol consumption pattern in the AQUA cohort was the ‘high-sustained’ drinking trajectory, which comprised fewer than 30 participants, thus limiting our ability to interpret our null finding with confidence.

The same meta-analysis also reported that PAE effects were influenced by socioeconomic status (SES) with the ’middle-class’ cohort of Seattle showing the smallest effect sizes^34^. Bingol et al. reported as early as 1987 that the risk of bearing a child with Fetal Alcohol Syndrome was 15.8 times higher for women with similar drinking levels if they were of lower SES^6,35^. Since then, other studies have shown that the risks for harm from PAE depend on a number of socio-environmental factors and cannot be completely attributed to the dose, frequency and timing of the exposure, a phenomenon aptly termed as ‘multidimensional’ by May and Gossage^6^.

Related to SES is parental educational attainment. Almost three quarters of AQUA mothers had completed a tertiary education, the highest proportions being in the moderate and high sustained drinking groups, with 74% and 80% respectively. We found that above all other factors considered in the regressions, maternal education most strongly influenced general cognition and academic skills in the children and that factors such as financial difficulties and parenting style also played a role. Thus, our null findings in this generally well-educated cohort may in part be explained by the concept of the multidimensional risk and taken together with the findings of the Seattle study^34^, any putative effects of PAE, at low to moderate levels at least, may have been substantially ameliorated by a favourable family environment. Thus, while we cannot rule out that low to moderate levels of PAE have an effect of children’s neuropsychological outcomes, the literature as a whole suggests that a child’s home environment plays an important role in whether any detrimental effects are measurable.

Our null findings contrast with a recent secondary analysis of 9,719 children from the Adolescent Brain Cognitive Development (ABCD) study, which reported that even children with low PAE demonstrated poorer psychological and behavioural outcomes at around 9–10 years of age^36^. The levels of PAE were somewhat similar to those in AQUA and the study considered potential confounding factors and applied demographic matching procedures for some of their analyses. However, while the ABCD study’s sample size is considerable, collection of all exposure and confounder information occurred retrospectively around 10 years after birth, raising questions around recall accuracy, especially for the short time prior to pregnancy recognition. It is likely that this could result in under-reporting of pregnancy alcohol consumption and therefore an overestimation of any PAE effects in the low to moderate range.

Further complicating any interpretation between PAE and child outcomes is that the toxic and teratogenic effects of PAE are complex and individually variable, due in part to various genetic contributions^37^. It is likely that PAE affects distinct aspects of fetal development by different mechanisms, which in turn are highly dependent on the timing of exposure. These include epigenetic processes and translational regulation and cytokine-mediated immune responses^38^.

Therefore, even having adjusted for relevant known confounding factors, as we did in the AQUA study, it may still be impossible to quantify the risk of harm from different levels and timing of PAE until we have a better understanding of the interaction between PAE, genetic, biological and environmental factors. This may require innovative research methodologies such as sibling design, mendelian randomisation and consideration of epigenetic mechanisms as mediators in the causal pathway.

Some methodological limitations potentially affecting the outcomes of this study need to be considered. First, exposure measurement was based on self-report. To maximise accurate reporting, the instrument for assessing PAE in the AQUA study underwent extensive development which included a literature review, expert consultations and consumer input (focus groups, pilot questions)^39^.

The study’s original recruiters were midwives experienced in engaging pregnant women and who received training on the study before commencing field work. They were able to spend time with potential participants to explain the details of the study as needed. The women were recruited from low-risk antenatal clinics and did not include those with alcohol and other drug dependence, where concerns for long-term child outcomes could be significant. Consequently, we expect the risk for stigma, guilt and blame resulting in exposure reporting bias to be low. Participants found the study’s aims very relatable, wishing to honestly contribute their experiences to advance knowledge and hopefully contribute to improved health advice for future pregnant women^40^.

Second, some of the outcome measures were obtained by parent report and it is possible that those who consumed moderate to high levels of alcohol assessed their children more favourably compared with mothers who consumed less alcohol (e.g. with some of the SDQ scores). However, a similar pattern of higher levels of performance was observed in aspects of some of the direct child assessments.

Third, attrition may have resulted in a biased selection of the current study sample. Controls were less likely to participate than those with PAE^21^. It is possible that mothers in the control group were more likely to take part if they felt there was a benefit for their child to have a neuropsychological assessment beyond the scope of the research, i.e. if there were some parental concerns about the child’s development. This may have contributed to somewhat lower outcome scores among controls but did not affect our interpretation of results. In conclusion, this study found no evidence that low to moderate or sporadic prenatal alcohol consumption was associated with adverse neurodevelopmental outcomes in early school-aged children. However, our findings have not established a safe level of alcohol consumption and may partly be explained by cumulative positive effects of favourable socio-environmental factors on child neurodevelopment. Abstinence in pregnancy continues to be the safest option.

### Technical considerations

The global pandemic disrupted recruitment and forced rapid adaptation of our neuropsychological assessments to telehealth-style administration initially, then later with physical distancing in place. Assessment protocols were adjusted to deal with covid restrictions. This meant that some sub-tests could not be administered for all children and the full-scale IQ index was computed using substitute sub-tests for one third of the children. There was also the potential for test scores to be differentially affected by testing modality. We considered this in our analyses where applicable (Supplementary Tables 2 and 3) but found no association between assessment type and outcome scores in the general cognition and academic skills domains.

### Supplementary Information

Supplementary Tables.

## Data Availability

The data that support the findings of this study are available from the corresponding author but restrictions apply to the availability of these data, which were used under license for the current study, and so are not publicly available. Data are however available from the authors after securing ethical permission and appropriate data access agreements.
